# Valorization of Carménère Grape Pomace: Extraction, Microencapsulation, and Evaluation of the Bioactivity of Polyphenols in Caco-2 Cells

**DOI:** 10.3390/ijms26167994

**Published:** 2025-08-19

**Authors:** Paula Valenzuela-Bustamante, Paula Cornejo, Nicolás Nolan, Alina Concepción-Alvarez, Raquel Bridi, Miguel Ángel Rincón-Cervera, Omar Porras, Adriano Costa de Camargo, M. Fernanda Arias-Santé

**Affiliations:** 1 Centro de Excelencia en Nanotecnología Leitat Chile, Fundación Leitat Chile, Román Díaz 532, Providencia, Santiago 8380000, Chile; pvalenzuela@leitat.cl (P.V.-B.); paulacornejo62@gmail.com (P.C.); nnmella@gmail.com (N.N.); 2 Instituto de Nutrición y Tecnología de los Alimentos, Universidad de Chile, El Líbano 5524, Macul, Santiago 8380000, Chile; a24ca90@gmail.com (A.C.-A.); marincer@inta.uchile.cl (M.Á.R.-C.); omar.porras@inta.uchile.cl (O.P.); 3 Departamento de Química Farmacológica y Toxicológica, Facultad de Ciencias Químicas y Farmacéuticas, Universidad de Chile, Santiago 8380000, Chile; raquelbridi@ciq.uchile.cl; 4 Departamento de Agronomía, Universidad de Almería, La Cañada de San Urbano s/n, 04120 Almería, Spain

**Keywords:** Carménère grape pomace, polyphenols, microencapsulation, Caco-2 cells, revalorization

## Abstract

Grape pomace is a major by-product of winemaking and a rich source of phenolic compounds with antioxidant potential. The Carménère variety, emblematic of Chilean viticulture, remains underutilized despite its high anthocyanin and flavanol content. This study aimed to develop a cost-effective method to recover and stabilize bioactive compounds from Carménère grape pomace. Five extracts were obtained using ethanol–water mixtures (0–100%) and characterized by HPLC-DAD and antioxidant assays (DPPH, FRAP, ORAC-FL). The 80% ethanol extract (EET-80) showed the highest antioxidant capacity (FRAP: 2909.3 ± 37.6; ORAC-FL: 1864.3 ± 157.8 µmol TE/g dw) and was selected for microencapsulation via spray drying using maltodextrin. This scalable technique protects thermosensitive compounds and enhances their applicability. The optimized 1:50 extract-to-carrier ratio achieved high encapsulation efficiency (85.7 ± 0.7%). In Caco-2 cells, the microencapsulated extract (5–250 µg/mL) showed no alteration in metabolic activity and significantly reduced intracellular ROS levels (65% inhibition at 250 µg/mL). Solvent polarity selectively influenced polyphenol recovery—50% ethanol favored catechin (581.1 µg/g) and epicatechin (1788.3 µg/g), while 80% ethanol enhanced malvidin-3-*O*-glucoside (118.0 µg/g). These findings support the valorization of Carménère grape pomace as a sustainable source of antioxidants and highlight the role of microencapsulation in improving extract stability and functionality.

## 1. Introduction

Wine consumption is widely observed around the world and has grown steadily over the past decades, establishing itself as one of the most popular and culturally significant alcoholic beverages globally. Particularly in Chile, the production of Carménère wine has experienced a remarkable resurgence since its rediscovery in the 1990s. Chile has become the world’s leading producer of this red wine, with approximately 95 million liters per year [1]. This success is attributed in part to Chile’s favorable climatic conditions; its terroir; the unique combination of soil, climate, topography, and human factors that shape the wine’s character [2]; and the absence of phylloxera, a pest that devastated European vineyards in the 19th century [1,3].

The production of Carménère wine generates around 22 million kg of grape pomace, an organic by-product composed of seeds, stems, and grape skins. This has become a significant environmental issue as it can lead to unpleasant odors, soil and water contamination from leaching pollutants, and greenhouse gas emissions [1,4]. Traditionally, these by-products were discarded or used in a limited way for composting and animal feed. However, their high content of bioactive compounds, such as polyphenols, has sparked growing interest in their valorization and utilization, transforming what was once waste into a valuable resource for various applications, ranging from the food industry to pharmaceuticals and cosmetics [5]. In addition, Carménère pomace is known to retain up to 60% of the original grape’s polyphenols, especially anthocyanins, flavanols, and phenolic acids [4].

The polyphenols present in wine and grape pomace, especially in red varieties, have garnered considerable interest within the scientific community due to their potential health benefits [6]. These compounds, which include flavonoids, resveratrol, and phenolic acids, possess potent antioxidant and anti-inflammatory properties [7]. Epidemiological studies and clinical trials suggest that moderate red wine consumption may be associated with a reduced risk of developing cardiovascular diseases thanks to the ability of polyphenols to improve endothelial function, reduce low-density lipoprotein (LDL) oxidation, and modulate blood pressure [8,9,10]. According to the U.S. Dietary Guidelines, moderate alcohol consumption is defined as up to one alcoholic drink per day for women and up to two drinks per day for men, where one drink-equivalent contains approximately 14 g (0.6 fl oz) of pure alcohol [11].

Particularly in Carménère grape pomace extracts, the most abundant compounds that have been identified include gallic acid, catechin, epicatechin, quercetin, and malvidin-3-*O*-glucoside, with concentrations varying depending on the extraction method and solvent system used [12,13]. Over the past decade, efforts have intensified to optimize eco-friendly extraction methods that recover these bioactives efficiently. In this context, ethanol–water mixtures are commonly used as green solvents due to their food-grade status and tunable polarity, which enables the selective extraction of both hydrophilic and lipophilic phenolics [12,14]. While some studies have explored hot pressurized liquid extraction (HPLE) or the use of deep eutectic solvents (DESs) [1,12,15], conventional atmospheric hydroalcoholic extraction remains a cost-effective and scalable strategy, especially when targeting small- and medium-sized industry applications.

In recent years, significant efforts have been made to develop and optimize technologies that improve the nutraceutical properties of dry powders, aiming to produce functional foods with enhanced bioavailability, stability, and shelf life. Among these, microencapsulation of polyphenols by spray drying has emerged as a promising and widely used technique in both food and pharmaceutical research due to its ability to enhance the stability and bioactivity of these sensitive compounds [16]. This method involves the atomization of a polyphenol-rich solution or emulsion, typically containing a carrier agent such as maltodextrin, into a hot-air chamber where the solvent evaporates rapidly, forming dry microcapsules. Spray drying offers several advantages, including protection of polyphenols from light, oxygen, and thermal degradation, thereby extending product shelf life and preserving antioxidant capacity [17]. Moreover, this technique allows controlled and targeted release of polyphenols in the gastrointestinal tract, which can enhance their absorption and biological efficacy. Recent studies have shown that spray-dried polyphenols exhibit improved functional properties compared to their free forms, maintaining higher antioxidant capacity and stability under storage conditions. These findings support the application of spray drying for the development of fortified food matrices and dietary supplements with improved health-promoting potential [16,18,19,20].

In this context, the present study aimed to develop a cost-effective and scalable methodology for the recovery and stabilization of natural antioxidant compounds from Carménère grape (*Vitis vinifera* L.) pomace. Five extracts were prepared using a conventional solid–liquid extraction method with different ethanol-to-water ratios (0%, 25%, 50%, 80%, and 100%) to evaluate the effect of solvent polarity on polyphenol recovery and antioxidant potential. The phenolic profile of the extracts was characterized by HPLC-DAD, and their antioxidant capacity was assessed using DPPH, FRAP, and ORAC-FL assays. The extract with the highest antioxidant performance (EET-80) was then microencapsulated by spray drying using maltodextrin as the encapsulating agent, aiming to enhance its stability and bioactivity.

Finally, to explore the biocompatibility and support the potential nutraceutical application of the encapsulated extract, we conducted in vitro cell-based assays using undifferentiated Caco-2 cells, a widely used model of the human intestinal epithelium. This approach enabled the preliminary evaluation of the metabolic activity and antioxidant effects of the spray-dried extract under physiologically relevant conditions.

## 2. Results

### 2.1. Carménère Grape Pomace Extraction

Table 1 presents the extraction yields obtained from Carménère grape pomace using dynamic maceration (50 °C, 90 min, 250 rpm) with ethanol–water mixtures of different proportions (0%, 25%, 50%, 80%, and 100% ethanol). The initial pH of the solvent systems ranged from 3.80 to 5.00 depending on ethanol content.

Extraction yields varied significantly among the solvent mixtures, ranging from 13.32% to 22.03%, relative to the initial dry mass. The highest yield was obtained using the EET-25 formulation (25% ethanol, pH 4.00), reaching 22.03 ± 0.05%, while the lowest yields were observed in the EGE (100% ethanol) and EGA (100% water) extracts, with 13.32 ± 0.81% and 13.75 ± 2.42%, respectively. Intermediate ethanol concentrations (particularly 25%) appeared to favor the solubilization of polar compounds, likely due to a more balanced solvent polarity.

Statistical analysis confirmed significant differences between EET-25 and the other treatments (*p* < 0.05), emphasizing the role of solvent composition in extraction efficiency. These results highlight the importance of optimizing solvent polarity to enhance the recovery of valuable compounds from agro-industrial residues such as grape pomace.

### 2.2. Effect of Water and Ethanol Percentage on the Extraction of Phenolic Compounds from Carménère Grape Pomace

#### 2.2.1. Total Phenolic Content (TPC), Total Flavonoid Content (TFC), Total Monomeric Anthocyanin Content (TMAC), and Condensed Tannin Content (CTC)

To recover phenolic compounds from Carménère grape pomace, we applied a conventional extraction method using hydroalcoholic mixtures with different ethanol-to-water ratios as solvents. Ethanol was selected as the solvent because it is generally recognized as safe (GRAS) for potential application in food products.

**Figure 1:**
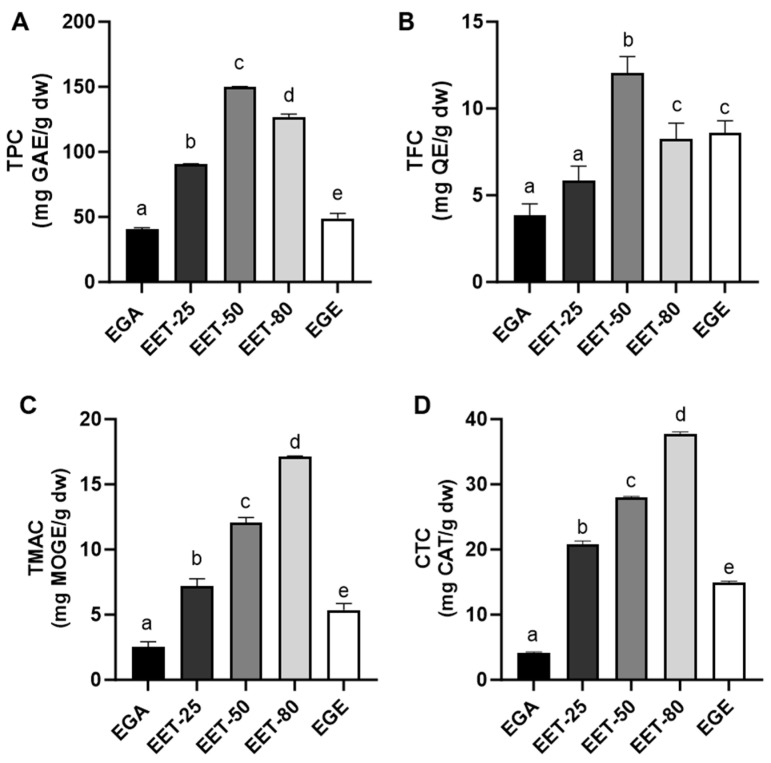
Characterization of grape pomace extracts from the Carménère variety obtained using different ethanol–water ratios. (**A**) Total phenolic content (TPC) is expressed as mg gallic acid equivalent (GAE)/g of dry extract. (**B**) Total flavonoid content (TFC) is expressed as mg quercetin equivalents (QE)/g of dry residue. (**C**) Total monomeric anthocyanin content (TMAC) is expressed as mg malvidin-3-*O*-glucoside equivalents (MOGE)/g of extract, and (**D**) condensed tannin content (CTC) is expressed as mg catechin equivalents (CAT)/g of extract. Different letters above the bars indicate statistically significant differences between groups, determined using one-way ANOVA followed by Tukey’s post hoc test (*p* < 0.05).

Figure 1 presents the results for total phenolic content (TPC), total flavonoid content (TFC), total monomeric anthocyanin content (TMAC), and condensed tannin content (CTC). The extract with the highest concentration of TPC and TFC was EET-50, with values of 149.91 ± 0.42 mg GAE/g dw and 12.06 ± 0.93 mg QE/g dw, respectively. For both compound groups, their concentration increased as the ethanol percentage rose to 50%. However, at higher ethanol proportions, an inverse effect was observed, leading to a decrease in recovered phenolic compounds. The extracts obtained with 100% water (EGA) and 100% ethanol (EGE) showed similar TPC concentrations (~4%), although EGE exhibited a higher concentration of flavonoid-type phenols.

Regarding other quantified phenolic compounds, an increase in TMAC was observed as the ethanol percentage increased, reaching a maximum at 80% *v*/*v*. The EGE-80 extract had the highest anthocyanin content, with 17.14 ± 0.04 mg MOGE/g dw. Once again, the global extracts (EGA and EGE) presented the lowest values, with 2.52 ± 0.40 and 5.35 ± 0.52 mg MOGE/g dw, respectively. A similar trend was observed for condensed tannins, whose concentration increased with ethanol content up to 80%. In this case, EGA exhibited the lowest value, at 4.16 ± 0.11 mg CAT/g dw.

#### 2.2.2. Polyphenolic Profile of the Extracts Determined by HPLC-DAD

The chemical characterization of the hydroalcoholic extracts from Carménère grape pomace by HPLC-DAD revealed significant differences in the polyphenolic profile depending on the type of solvent used. The results for the identified phenolic compounds in each extract are summarized in Table 2.

The EET-50 extract exhibited the most balanced and enriched profile of bioactive compounds among all tested formulations. It showed the highest concentrations of gallic acid (578.89 ± 7.02 µg/g dw), catechin (581.12 ± 30.09 µg/g dw), epicatechin (1788.33 ± 218.66 µg/g dw), and kaempferol-3-rutinoside (0.568 ± 0.16 µg/g dw), highlighting the effectiveness of 50% ethanol as a solvent for recovering both polar and semi-polar phenolic compounds. These findings suggest that a 1:1 ethanol–water mixture enhances the solubilization and diffusion of secondary metabolites from the grape pomace matrix, likely due to its optimal polarity.

The EET-80 extract also demonstrated a high phenolic content, although slightly lower than EET-50 for catechin (437.11 ± 16.31 µg/g dw) and epicatechin (1365.41 ± 107.15 µg/g dw). However, it contained the highest level of malvidin-3-*O*-glucoside (118.04 ± 4.68 µg/g dw), an anthocyanin largely responsible for the characteristic red color of grapes and wine derivatives. This may be attributed to the greater affinity of anthocyanins for less polar solvents such as ethanol at high concentrations.

In contrast, the EET-25 extract showed an intermediate extraction efficiency. Although it contained more phenolic compounds than the aqueous extract (EGA), its levels were markedly lower than those of EET-50 and EET-80. Catechin and epicatechin were quantified at 474.47 ± 41.28 and 1361.11 ± 80.43 µg/g dw, respectively, and malvidin-3-*O*-glucoside reached 66.33 ± 2.22 µg/g dw, confirming the lower efficiency of 25% ethanol for extracting less polar flavonoids.

As expected, the aqueous extract (EGA) presented the poorest phenolic profile, with notably low concentrations of catechin (34.15 ± 1.16 µg/g dw), epicatechin (71.43 ± 5.89 µg/g dw), and malvidin-3-*O*-glucoside (27.19 ± 1.59 µg/g dw). While water is a safe and sustainable solvent, its limited ability to dissolve medium- and low-polarity compounds restricts its effectiveness for phenolic extraction in this context.

Interestingly, the global ethanolic extract (EGE)—despite having the highest concentrations of gallic acid (587.22 ± 14.74 µg/g dw) and epicatechin (2670.36 ± 114.54 µg/g dw)—exhibited a less diverse composition. Catechin and malvidin-3-*O*-glucoside were substantially lower (69.90 ± 6.66 and 15.16 ± 0.68 µg/g dw, respectively) compared to EET-50 and EET-80.

In addition to the major polyphenols, several minor but bioactive compounds were identified, further differentiating the extracts. Vanillic acid was detected exclusively in EET-50 (216.33 ± 1.25 µg/g dw), underscoring its unique composition. Kaempferol was found mainly in EET-25, EET-50, and EET-80 (up to 0.097 ± 0.01 µg/g dw), as was kaempferol-3-rutinoside, suggesting that intermediate ethanol concentrations favor flavonol extraction. Quercetin was present in all extracts, but at the highest levels in EET-80 (0.66 ± 0.02 µg/g dw) and EET-50 (0.60 ± 0.01 µg/g dw). Minor flavan-3-ols such as epigallocatechin gallate and epicatechin gallate were also found in small amounts, primarily in EET-50 and EGE. These findings confirm that solvent polarity plays a key role in selectively recovering specific phenolic subclasses, reinforcing the superior compositional quality of EET-50 and EET-80.

### 2.3. Antioxidant Capacity of the Carménère Grape Pomace Extracts

The antioxidant capacity of the extracts from Carménère grape pomace showed a strong dependence on the solvent composition, as reflected by the variations observed across the DPPH, FRAP, and ORAC-FL assays (Table 3). The EET-50 exhibited the highest antioxidant capacity in the DPPH assay (985.03 ± 47.04 µmol TE/g dw), followed closely by EET-80 (884.43 ± 41.31 µmol TE/g dw). Similar trends were observed in the ORAC-FL and FRAP assays, where EET-80 showed the highest ferric reducing ability (FRAP = 2909.33 ± 37.55 µmol TE/g dw), while both EET-50 and EET-80 presented a strong peroxyl radical scavenging capacity.

In addition to absolute antioxidant capacity, the inhibitory concentration required to reduce 50% of DPPH radicals (IC_50_) provides a comparative measure of the potency of each extract. The results revealed that EET-50 and EET-80 were the most potent extracts, exhibiting the lowest IC_50_ values of 17.96 ± 0.87 µg/mL and 20.00 ± 0.92 µg/mL, respectively. These values are consistent with their high levels of phenolic compounds and strong antioxidant responses in all assays. In contrast, EGA required a much higher concentration to achieve the same radical inhibition effect (IC_50_ = 229.60 ± 8.75 µg/mL), highlighting its comparatively weaker antioxidant potency. The intermediate IC_50_ values observed for EET-25 and EGE (approximately 50 µg/mL) further support the notion that both the quality and the balance of phenolic compounds within the extract are critical for maximizing antioxidant efficiency.

To better understand the relationship between phenolic composition and antioxidant capacity, a Pearson correlation analysis was performed using total phenolic content (TPC), selected individual phenolics (e.g., catechin, epicatechin, gallic acid, and malvidin-3-*O*-glucoside), and antioxidant values. Among the compounds analyzed, malvidin-3-*O*-glucoside exhibited a strong and statistically significant correlation with the DPPH values (r = 0.89, *p* = 0.042) and ORAC-FL (r = 0.91, *p* = 0.032), confirming its important role in free radical scavenging. This aligns with the profile of the EET-80 extract, which had the highest anthocyanin content and the strongest FRAP and ORAC responses.

Likewise, positive correlations were observed between the DPPH values and both TPC (r = 0.78) and catechin (r = 0.79), although these were not statistically significant (*p* > 0.05). Nevertheless, this trend is consistent with the high performance of the EET-50 extract, which presented the highest TPC (149.91 ± 0.42 mg GAE/g dw), high catechin levels, and the best DPPH antioxidant response. In contrast, epicatechin, despite being highly abundant in EGE, showed no significant correlation with antioxidant capacity (r < 0.38 in all assays), suggesting that its contribution may depend on interactions with other phenolics.

Overall, the findings indicate that antioxidant capacity is not solely determined by the presence of individual compounds but by the synergistic interaction among multiple phenolic constituents. The simultaneous presence of flavonoids, phenolic acids, and anthocyanins—especially in the EET-50 and EET-80 extracts—appears to be essential to maximize antioxidant functionality.

### 2.4. Microencapsulation of Carménère Pomace Grape Extract

To evaluate the protection and stabilization of bioactive compounds—particularly polyphenols with a high antioxidant capacity—found in Carménère grape pomace under adverse environmental conditions such as light, oxygen, and humidity, microencapsulation of one of the extracts was carried out using spray drying.

The EET-80 extract was selected for microencapsulation due to its highest antioxidant capacity (as determined by FRAP and ORAC-FL assays) among the evaluated extracts. Maltodextrin (MD) was chosen as the encapsulating matrix because it is widely used in techniques such as spray drying, owing to its favorable physicochemical properties. Its high water solubility, low viscosity at high concentrations, and good film-forming ability enable the efficient encapsulation of bioactive compounds, protecting them from environmental factors such as light, oxygen, and humidity. Moreover, maltodextrin is tasteless, colorless, and inexpensive, facilitating its incorporation into food, cosmetic, and pharmaceutical formulations without negatively affecting the sensory characteristics of the final product. It also acts as a physical barrier, contributing to the thermal and oxidative stability of the encapsulated compounds, making it a versatile and functional matrix for protecting antioxidant-rich extracts [21].

**Figure 2:**
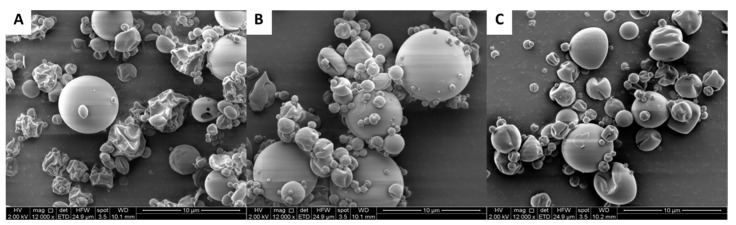
SEM micrographs of EET-80 microcapsules with different extract/maltodextrin ratios. (**A**) 1:10, (**B**) 1:50, and (**C**) 1:100. The SEM micrographs of the microcapsules were obtained at a magnification of 12,000× to allow detailed observation of their surface morphology.

The SEM micrographs shown in Figure 2 depict the morphology of the microcapsules obtained via spray drying of the antioxidant EET-80 extract from Carménère grape pomace using different extract/maltodextrin (MD) ratios. In Figure 2A (1:10), a heterogeneous mixture of particles is observed, many of which exhibit rough, collapsed, or irregular surfaces, suggesting low efficiency in forming spherical microcapsules and limited structural protection. In Figure 2B (1:50), the microcapsules appear predominantly spherical and uniformly sized, with smooth and well-defined surfaces, indicative of improved encapsulation and film formation by the coating agent. Finally, in Figure 2C (1:100), the microcapsules also show a mostly spherical morphology and smooth surface; however, some partially collapsed or deformed structures are noticeable, possibly due to the high proportion of maltodextrin, which may cause stress during the drying process. Overall, these results suggest that the 1:50 ratio provided optimal conditions for the formation of microcapsules with desirable morphological characteristics for enhanced stability and functionality.

Table 4 presents the physicochemical characterization of the microencapsulated EET-80 extract using different extract-to-maltodextrin (extract/MD) ratios. The encapsulation yield ranged from 73.56% to 82.34%, with the highest value observed in the 1:100 ratio. All formulations showed a microencapsulation efficiency (ME) above 80%, with the 1:10 and 1:50 ratios reaching 87.47% and 85.72%, respectively.

Particle size distribution analysis revealed Dx(90) values between 3.31 and 5.14 µm, with no statistically significant differences among the formulations (*p* > 0.05).

Color parameters were measured using the CIE Lab* system. The L* value (lightness) increased with higher maltodextrin content, indicating lighter powders. Meanwhile, the a* (green–red component) and b* (blue–yellow component) values progressively decreased, suggesting a shift toward less saturated and more neutral tones. These variations are consistent with the dilution of pigments in the extract as the proportion of the encapsulating matrix increases. This trend was also visually reflected in the color transformation from an intense purplish tone in lower maltodextrin ratios to a more attenuated and pale hue as the carrier concentration increased. To facilitate visual interpretation, the CIE Lab* values were converted to RGB color representations and are included in Table 4 as visual references.

### 2.5. Cellular Metabolic Activity and Antioxidant Capacity in Caco-2 Cells of Microcapsules

The microencapsulated powder of EET-80 from Carménère grape pomace that showed the best combination of high encapsulation yield (81.54%) and high encapsulation efficiency (85.77%) was the powder microencapsulated with maltodextrin at a 1:50 ratio of extract to coating matrix. Due to these favorable results, this formulation was selected for further evaluation in cellular assays.

**Figure 3:**
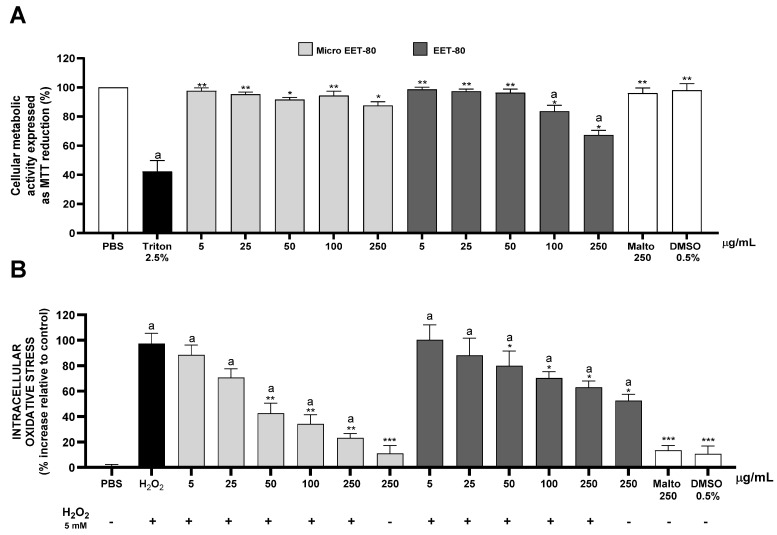
(**A**) Comparison of cellular metabolic activity, expressed as the percentage of MTT reduction inhibition, using 2.5% Triton as a positive control for cell death. (**B**) Cellular antioxidant capacity assessed by the intracellular oxidation of the fluorescent probe DCHF-DA using 5 mM H_2_O_2_ as an oxidative stress inducer. Abbreviations: Malto—maltodextrin. In both assays, the treatments evaluated were the following: (Micro EET-80) EET-80 microencapsulated with Malto at a 1:50 ratio, and EET-80 resuspended in 0.5% DMSO and added directly to Caco-2 cells. These treatments were applied at concentrations ranging from 5 to 250 µg/mL for 40 min. Additionally, Malto at 250 µg/mL was evaluated as the encapsulating polymer control, and 0.5% DMSO as the solvent used to resuspend EET-80. Significant differences as determined by one-way ANOVA followed by Tukey’s post hoc test were considered to be * *p* < 0.05, ** *p* < 0.005, and *** *p* < 0.001 in relation with cells treated with Triton or hydrogen peroxide, while for “a”, it was considered *p* < 0.005 in relation to cells treated with PBS (*n* = 3).

Figure 3 presents the results of cellular metabolic activity (A) and cellular antioxidant capacity (B) in Caco-2 cells treated with the ethanolic extract of Carménère grape pomace (EET-80), either in its microencapsulated form with maltodextrin (Micro EET-80) or in its free form (EET-80), at concentrations ranging from 5 to 250 µg/mL for 40 min.

Figure 3A shows the effect of the different treatments on metabolic activity of Caco-2 cells, expressed as the percentage inhibition of MTT reduction, an indirect indicator of mitochondrial activity and, consequently, an estimation of cytotoxicity. Treatment with Micro EET-80, corresponding to the total ethanolic extract of Carménère grape pomace microencapsulated with maltodextrin at a 1:50 ratio, did not produce significant changes in cell metabolic activity across the entire concentration range tested (5 to 250 µg/mL), with inhibition percentages similar to the negative control (PBS-treated cells). This indicates the good biocompatibility of the microencapsulated extract, even at the highest concentration evaluated.

In contrast, the free EET-80 extract (dissolved in 0.5% DMSO and applied directly to the cells) showed a slight trend toward reduced MTT reduction inhibition at higher concentrations (≥100 µg/mL). This suggests a possible mild cytotoxic effect attributable to the nonencapsulated extract, potentially associated with greater direct exposure to phenolic compounds. The individual controls, maltodextrin (Malto 250) and 0.5% DMSO, did not show significant deleterious effects, confirming that the effects observed in the active treatments were mainly due to the delivery form of the extract and not to the encapsulating matrix or solvent. The positive control (cells treated with 2.5% Triton) showed a marked decrease in MTT reduction, validating the assay.

Taken together, these results suggest that microencapsulation of the extract not only preserves its safety but also reduces the potential adverse effects on metabolic activity observed with the free extract.

Figure 3B shows the cellular antioxidant capacity of the treatments, measured by the inhibition of intracellular oxidation of the fluorescent probe DCHF-DA induced by hydrogen peroxide (H_2_O_2_ 500 µM) in Caco-2 cells. The microencapsulated extract (Micro EET-80) exhibited significant inhibition of H_2_O_2_-induced fluorescence starting at 100 µg/mL, with a particularly strong effect at 250 µg/mL, where a substantial reduction in intracellular oxidative stress was observed compared to the H_2_O_2_-treated control. This response suggests that the bioactive compounds present in the extract are capable of neutralizing reactive oxygen species (ROS) at the cellular level, likely due to improved stability and bioavailability facilitated by the microencapsulation process.

In contrast, the free extract (EET-80) did not produce a significant antioxidant effect at any of the concentrations tested, with fluorescence levels remaining comparable to those of the H_2_O_2_ control. This lack of effect may be attributed to the lower stability of phenolic compounds in aqueous solution or limited cellular uptake due to the absence of protection and delivery mechanisms provided by microencapsulation.

The maltodextrin and DMSO controls did not show a significant antioxidant capacity, confirming that the observed effect in Micro EET-80 is attributable to the action of the antioxidant compounds in the extract. Overall, these results highlight the importance of microencapsulation in preserving or enhancing the antioxidant functionality of the extract in a cellular model.

## 3. Discussion

Carménère grape pomace has gained increasing attention in recent years due to its unique phenolic composition, which includes high concentrations of anthocyanins (notably malvidin-3-*O*-glucoside), flavonols, and flavanols such as catechin and epicatechin. These compounds are recognized for their antioxidant, anti-inflammatory, and health-promoting properties, and their abundance in the pomace matrix makes this by-product a promising source of natural bioactive ingredients for food and nutraceutical applications [22].

The extraction yields obtained in this study are consistent with those previously reported for grape pomace and other wine industry by-products, where the efficiency of recovery is highly influenced by the type of solvent and extraction method used. For instance, Jara-Palacios et al. [23] reported extraction yields ranging from 17% to 30% (*w*/*w*) when applying conventional maceration (1 h, room temperature) to pomaces from different grape varieties. Their study found the highest yield with 20% ethanol, suggesting a preferential extraction of more polar compounds such as phenolic acids and tannins. This observation aligns with the findings of Spigno et al. [24], who highlighted that increasing water content in hydroalcoholic mixtures enhances the extraction of polar phenolic compounds. In our study, the highest extraction yield was obtained using 25% ethanol, reaching 22.03 ± 0.05%. In contrast, the use of higher ethanol concentrations (≥50%) resulted in a decrease in extraction yield. For example, the EET-50 and EET-80 extracts showed yields of 15.10% and 13.67%, respectively. These values are comparable to those reported by López-Astorga et al. [25], who obtained a 9.59 ± 0.65% yield using 68% ethanol in a 1 h extraction at 30 °C from Cabernet Sauvignon grape pomace. In their study, the extract was further used to produce microcapsules that were incorporated as antioxidant ingredients into yogurt formulations.

Despite their lower yields at higher ethanol concentrations, the extracts obtained with 50% and 80% ethanol exhibited the highest total phenolic content and antioxidant capacity, which will be addressed below.

Our results are consistent with the literature regarding the phenolic fingerprint of Carménère grape pomace. Extracts obtained with 50% and 80% ethanol were enriched in malvidin-3-*O*-glucoside, catechin, epicatechin, and gallic acid—compounds that are typically abundant in Carménère skins and seeds and contribute to their intense pigmentation and biological activity [13]. Notably, the content of total anthocyanins and condensed tannins increased with higher ethanol concentrations, peaking at 80% ethanol. These observations closely match the results reported by Jiménez-Moreno et al. [26], who identified 50% ethanol as optimal for total phenolics and 75% ethanol for flavonoids in grape stem extracts. Their findings support our conclusion that ethanol concentration is a critical factor in maximizing both phenolic extraction and antioxidant capacity.

Furthermore, previous studies using hot pressurized liquid extraction (HPLE) have shown that Carménère pomace retains up to 60% of its original polyphenol content after vinification, especially in the form of proanthocyanidins and monomeric flavanols [4]. Although our methodology relied on atmospheric solid–liquid extraction, the phenolic profiles and antioxidant capacities of our EET-50 and EET-80 extracts were comparable or superior to those obtained using HPLE. For instance, Huamán-Castilla et al. [1] showed a moderate antioxidant capacity in Carménère pomace extracts obtained with aqueous deep eutectic solvents (DESs), reporting DPPH values between 93.17 and 140.39 µmol TE/g dry pomace and ORAC values between 130 and 140 µmol TE/g. In contrast, our EET-50 and EET-80 extracts showed significantly higher antioxidant capacities (ORAC-FL: 1571.26 and 1864.32 µmol TE/g dw, respectively), indicating that properly optimized ethanol–water extractions under atmospheric conditions may rival or outperform more energy-intensive extraction techniques.

This enhanced performance is not only due to solvent optimization but also likely stems from a favorable balance among polyphenolic families in the extracts. Malvidin-3-*O*-glucoside—a compound repeatedly associated with a strong antioxidant capacity—was most abundant in the EET-80 extract and has been highlighted as a key contributor to bioactivity in grape-derived products [13,27,28].

Regarding the stabilization of bioactivity, the microencapsulation of EET-80 by spray drying with maltodextrin produced powders with distinct morphological and functional characteristics depending on the extract-to-carrier ratio. SEM analysis revealed that low maltodextrin content (1:10) led to irregular and partially collapsed particles, indicating poor structural integrity. Conversely, the 1:50 formulation yielded spherical, smooth-surfaced microcapsules, reflecting effective film formation and encapsulation. Although a higher maltodextrin ratio (1:100) improved drying yield, it also introduced signs of structural collapse due to excess carrier. Both encapsulation yield and efficiency increased with the maltodextrin ratio, peaking at 61.15% and 87.52%, respectively, for the 1:100 and 1:50 formulations. However, increasing the carrier concentration reduced the phenolic content per gram of powder, underlining the need to balance protection and potency for application-specific goals. As expected, antioxidant capacity declined with higher maltodextrin content due to dilution of the active extract. Nevertheless, the 1:50 ratio emerged as the optimal formulation, combining high encapsulation efficiency with substantial antioxidant capacity—an outcome consistent with previous studies on microencapsulation of phenolic compounds [26].

The enhanced cellular metabolic activity and antioxidant capacity observed with Micro EET-80 align closely with the existing literature on phenolic compound stabilization via maltodextrin microencapsulation [29,30]. In our study, encapsulated EET-80 did not induce any alteration in metabolic activity (assessed via MTT reduction) in Caco-2 cells at concentrations up to 250 µg/mL, whereas the nonencapsulated extract caused a slight reduction in metabolic activity. This finding is consistent with previous research showing that green tea catechins encapsulated in maltodextrin exhibited greater cellular compatibility compared to their free form [31].

The observed biological effects of Micro EET-80 on Caco-2 cells can be directly attributed to the main phenolic compounds identified in the extract by HPLC-DAD, particularly malvidin-3-*O*-glucoside, epicatechin, catechin, and gallic acid. Malvidin-3-*O*-glucoside, the predominant anthocyanin in EET-80, has been widely documented for its intracellular antioxidant capacity and membrane-stabilizing properties, which could explain the significant reduction in oxidative stress observed in the H_2_O_2_-induced Caco-2 model [32,33]. Similarly, catechin and epicatechin are powerful radical scavengers with low cytotoxicity, and they have been shown to modulate oxidative stress pathways and support mitochondrial integrity in intestinal epithelial models [34,35]. The negligible effect of the free extract on cellular antioxidant response may be due to poor compound stability, degradation in solution, or limited bioavailability, all of which were likely mitigated by encapsulation.

Caco-2 cells are widely used in in vitro models for studying intestinal absorption and cytocompatibility of dietary compounds [36,37]. Upon culture to confluence and differentiation, they develop enterocyte-like features such as brush border enzymes, tight junctions, and polarized transport, making them more physiologically representative of the intestinal epithelium. This approach is particularly useful for detecting early adverse effects and evaluating metabolic activity in response to bioactive substances [38,39]. However, in this study, undifferentiated Caco-2 cells were used, which maintain a highly proliferative state and are particularly useful for detecting early cytotoxic effects and assessing changes in cellular metabolic activity in response to bioactive compounds.

This model allows for the identification of potential adverse effects at early stages but lacks the structural and functional complexity of the mature intestinal barrier. Therefore, we acknowledge that the use of undifferentiated cells represents a limitation in terms of mimicking the full physiological context of the human intestine and may reduce the translational relevance of the observed antioxidant effects [40].

To strengthen the interpretation of the results and enhance their applicability, future studies should incorporate differentiated Caco-2 monolayers, 3D intestinal co-culture systems, and in vivo models [41]. These approaches would allow for a more comprehensive evaluation of the bioavailability, transport, and protective effects of Carménère grape pomace polyphenols in physiologically relevant environments.

## 4. Materials and Methods

### 4.1. Standards and Reagents

The Folin–Ciocalteu reagent, sodium carbonate, 6-hydroxy-2,5,7,8-tetramethylchroman-2-carboxylic acid (Trolox), aluminum chloride, ferric chloride hexahydrate, Triphenyl tetrazolium chloride (TPTZ), 2,2-Diphenyl-1-picrylhydrazyl (DPPH), 2,2′-Azobis(2-methylpropionamidine) dihydrochloride (AAPH), fluorescein, standards for HPLC-DAD (catechin, epicatechin, epicatechin gallate, epigallocatechin gallate, gallic acid, kaempferol, kaempferol 3-rutinoside, malvidin-3-*O*-glucoside, procyanidin b1, quercetin, taxifolin, and vanillic acid), 2′,7′-dichlorofluorescein-diacetate (DCFH-DA), and 3-(4,5-dimethylthiazol-2-yl)-2,5-diphenyltetrazolium bromide (MTT) were all purchased from Sigma-Aldrich (Steinheim, Germany). Ethanol (EMSURE^®^ (Harrow, UK) Ethanol, ACS, ISO, Reag. Ph Eur, purity ≥ 99.9%) was purchased from Merck (Darmstadt, Germany).

### 4.2. Grape Pomace Sample

The Carménère grape pomace (*Vitis vinifera* L. var. Carménère) was generously provided by Viña Concha y Toro, located in the Pencahue Valley, Maule Region, Chile (35°25′36″ S 71°40′18″ W). The pomace was collected in 2022 after the winemaking process and consisted of a mixture of seeds, stems, and grape skins, from which the stems were separated. The sample was frozen at −20 °C and ground using a food processor (Waring^®^, (Stamford, CT, USA) model MX1200XTX, E.E.U.U) for subsequent extraction. The Carménère grape pomace exhibited a natural moisture content of approximately 60% at the time of collection.

### 4.3. Preparation of Carménère Grape Pomace Extracts

For the recovery of antioxidant compounds from grape pomace, a conventional extraction methodology using dynamic maceration [42] was selected. Different ethanol–water ratios were used to compare the chemical composition and antioxidant capacity of the obtained extracts. Five ethanol–water mixtures (0, 25, 50, 75, and 100%, *v*/*v*) were used for extraction, resulting in five dry extracts labeled as EGA, EET-25, EET-50, EET-80, and EGE, respectively.

For each extract, a solid-to-liquid (S/L) ratio of 1:4 was used, along with a constant temperature of 50 °C and stirring at 350 rpm for 90 min. After this time, the liquid was separated from the solid for each extract by vacuum filtration. The macerated liquid was transferred to a flask, and the solvent was removed using a rotary evaporator at 120 rpm and a temperature of 35 °C. Subsequently, the concentrated extracts corresponding to EGA, EET-25, EET-50, and EET-80 were reconstituted with 20 mL of Milli-Q water, transferred to a 50 mL tube, frozen at −20 °C, and lyophilized. Meanwhile, the concentrated EGE extract was reconstituted with 20 mL of ethanol (>99.9%) and dried in a vacuum oven at 35 °C.

The dry extracts were stored at 8 °C in amber-colored containers, and the yield percentage of each extract was calculated using the following equation:

(1) $$Yield\left(\%\right)\left(dry\;mass\right)=\frac{\left(g\right)\;dry\;extract\;mass\;\times 100}{\left(g\right)\;initial\;mass\;of\;pomace\;solids}$$

### 4.4. Total Phenolic Content (TPC), Total Flavonoid Content (TFC), Total Monomeric Anthocyanin Content (TmA), and HPLC Analyses

TPC method. Total phenolic content was analyzed using the Folin–Ciocalteu method described by Singleton et al. [43], with some modifications. Briefly, 100 µL of the extracts diluted in 40% *v*/*v* ethanol was taken and mixed with 100 µL of the 10% *v*/*v* F-C reagent. The mixtures were incubated at 40 °C for 2 min, in the dark, and 800 µL of 5% *w*/*v* Na_2_CO_3_ was added. The samples were incubated for 20 min at 40 °C and the absorbance of the samples was read at 760 nm in a UV-vis spectrophotometer (Shimadzu, model 2600i, Tokyo, Japan), using the 40% *v*/*v* ethanolic extract as a control and 40% *v*/*v* ethanol as a blank. The absorbance of the samples (*n* = 3) was interpolated on a calibration curve with gallic acid at concentrations between 10 and 80 µg/mL, which was performed with the same experimental protocol (y = 0.0101 x + 0.0126, R^2^ = 0.9997). The results were expressed in mg of gallic acid equivalents (mg GAE) per gram of dry extract weight (g dw).

TFC method. The flavonoid content (flavones and flavonols) was determined by a colorimetric method with AlCl_3_ in acidic medium according to Chang et al. [44]. For this purpose, 100 µL of the extracts dissolved in 80% *v*/*v* ethanol was mixed with 20 µL of a 10% *w*/*v* AlCl_3_ solution, 20 µL of 1 M CH_3_COONa, and 560 µL of Milli-Q water. The mixtures were incubated for 40 min at 25 °C and the absorbance was read at 415 nm in a UV-vis spectrophotometer (Shimadzu, model 2600i, Japan), using the 80% *v*/*v* ethanolic extract as a control and 80% *v*/*v* ethanol as a blank. The absorbances of the samples (*n* = 3) were interpolated on a calibration curve using quercetin as standard at concentrations between 10 and 80 µg/mL (y = 0.006 x + 0.0254, R^2^ = 0.9991). The results were expressed in mg of quercetin equivalents (mg QE) per gram of dry extract weight (g dw).

TMAC method. The total monomeric anthocyanin (TMAC) content was determined using the method described by Lee et al. [45], which is based on the color changes that these polyphenols present when in solution at different pH. The extracts were solubilized in 50% ethanol and a 25 µL aliquot was mixed with 975 µL of a 0.025 M KCl buffer to obtain the solution at pH 1. On the other hand, 25 µL of the solubilized extracts was mixed with 975 µL of a 0.4 M sodium acetate buffer to obtain the solution at pH 4.5. Both solutions were measured using a UV-vis spectrophotometer (Shimadzu, model 2600i, Japan) to obtain the absorbance at the maximum wavelength (A_λvis-max_) and at a wavelength of 700 nm (A_700_). Finally, the differential absorbance value (A) was obtained from the results of both measurements according to the following formula:

A = (A_527nm_ − A_700nm_)_pH 1.0_ − (A_527nm_ − A_700nm_)_pH 4.5_ (2)

Subsequently, the TMAC was determined according to the following formula:

TMAC (mg/L) = (A × MW × DF × 1000)/(ε × 1)(3)

where

A = Abs difference at different pH;
MW = Molecular weight of Malvidin-3-*O*-Glucoside;
ε = Molar absorptivity;
DF = Dilution factor;
1 = standard width of the cuvette (cm).

The results were expressed in mg of malvidin-3-*O*-glucoside equivalents (MOGE) per gram of dry extract weight (g dw).

CTC method. Condensed tannins were analyzed colorimetrically according to the method of Price, Hagerman, and Butler [46]. To 0.2 mL of methanolic solution of the resuspended extracts (10 mg) was added 5 mL of 0.5% vanillin reagent; a 5 mL volume of 4% concentrated HCl in methanol was used as a blank. The absorbances of the samples and the blank were read at 500 nm after standing for 20 min at room temperature. Catechin was used as a standard in these experiments. The condensed tannin content was expressed as mg of catechin equivalents per gram of dry weight of extract (DW).

HPLC analyses. Quantification of phenolic compounds in grape pomace extracts was performed using high-performance liquid chromatography coupled to a diode array detector (HPLC-DAD) (Hitachi Chromaster, 5000 series, Tokyo, Japan) according to the method described by Rivera et al. [47]. The HPLC was equipped with an autosampler and a diode array detector controlled by Chromaster System Manager V1.2 analysis software. Phenolic compounds were separated on a RP-18 Purospher STAR column (250 × 4.6 mm (inner diameter)) at 35 °C and a flow rate of 0.8 mL/min. Elution was carried out by gradient with the mixture of three mobile phases: (A) metanol, (B) acetonitrile and (C) formic acid (0.1%). The gradient program used was as follows: 0–10 min (20% B, 80% C), 10.1–40 min (7.5% A, 25% B, 67.5% C), 40.1–50 min (15% A, 25% B, 60% C), 50.1–65 min (15% A, 45% B, 40% C), and 65.1–80 min (20% B, 80% C). The injection volume was 10 μL and the absorbance was measured between 210 and 550 nm. Phenolic compounds were detected based on their UV–Vis spectrum and the retention time of each standard. Quantification was performed using the calibration curves of each standard in a range between 5 and 200 μM.

### 4.5. In Vitro Antioxidant Capacity

Three methods were used to evaluate the antioxidant potential of the five Carménère pomace extracts.

FRAP assay. The ferric reducing antioxidant power (FRAP) was determined according to the methodology of Benzie and Strain [48] with some modifications. For this purpose, 1500 μL was mixed with 200 μL of the extracts dissolved in methanol. The mixture was incubated at 37 °C for 60 min, the absorbance was read at 594 nm using a UV-Vis spectrophotometer (Shimadzu, model 2600i, Japan), and the results were interpolated on a calibration curve based on Trolox (25–250 μM/L). The FRAP value results were expressed as μmol equivalents of Trolox per g of dry extract weight (g dw) (μmol ET/g dw).

DPPH assay. The antioxidant capacity of the grape pomace extracts was evaluated using the DPPH assay described by Gonzales et al. [49]. This radical has a maximum absorbance at 517 nm, which disappears with the reduction of the antioxidant compound. The DPPH solution was prepared fresh at 0.1 mM, and 3 mL of this solution was mixed with 1 mL of the extracts diluted in methanol at a concentration of 50 ppm. The mixture was incubated for 30 min in the dark at room temperature and the absorbance was measured at 517 nm using a UV-Vis spectrophotometer (Shimadzu, model 2600i, Japan). The control consisted of the DPPH solution mixed with 1 mL of methanol, and its absorbance was used in the following equation to calculate the percentage inhibition of each extract:

(4) $$DPPH\;Inhibition\;\left(\%\right)=\frac{\left(Abs\;Control-Abs\;Extract\right)}{Abs\;Control}\times 100$$

Different concentrations of each extract were evaluated to obtain a % Inhibition vs. concentration curve. From this curve, the inhibitory concentration 50 (IC_50_) was calculated by logarithmic adjustment using the GraphPad Prism program version 10.0.0. The IC_50_ value was expressed in µg/mL.

ORAC-FL assay. Oxygen radical absorbance capacity (ORAC) was performed according to De Camargo et al. [50] with slight modifications. The reaction mixture consisted of 75 mM sodium phosphate buffer (pH 7.4), 70 nM fluorescein, 9 mM AAPH, and 20 µL of GP extract. Trolox (0–10 μM) was used as a standard for the calibration curve. The reaction was incubated for 20 min at 37 °C in the microplate reader (Infinite pro-200, Austria GmbH, Groedig, Austria) and the fluorescence decay over time was recorded (Ex: 480 nm and Em: 510 nm). Data were reported as μmol Trolox equivalent per gram of dry extract weight (μmol TE/g dw).

### 4.6. Spray Drying Process and Physicochemical Characterization of Microcapsules

A lab-scale Mini Spray Dryer B-290 (Büchi Labortechnik AG, Flawil, Switzerland) equipped with a 0.7 mm nozzle was used for obtaining the microencapsulated maltodextrin (MD) of a selected grape pomace extract (EET-80). The extract/carrier ratios varied between 1:10, 1:50, and 1:100. The operating parameters of the equipment were kept constant throughout the experiments: aspiration rate 32.5 m^3^/h, air flow 536 L/h, feed flow 3.0 mL/min, and equipment inlet temperature 160 °C. The samples were constantly agitated during the drying process to achieve a correct dissolution of the carrier. The color of the spray-dried powders was determined in triplicate (*n* = 3) using a WR10QC portable colorimeter (FRU, China), following the CIE Lab* color system. The measured coordinates (L*, a*, b*) were converted to RGB using the Color Designer online tool (https://colordesigner.io/convert/labtorgb, accessed on 30 July 2025), and the corresponding color representations were included as visual references. In addition, the particle size distribution of the three microencapsulated formulations was analyzed by laser diffraction using a Mastersizer 3000 instrument (Malvern Panalytical Ltd., Worcestershire, UK). The Dx(90) parameter (µm), representing the particle diameter below which 90% of the total analyzed volume is distributed, was used as the characteristic value for size comparison.

Total bioactive compounds (TBC), surface bioactive compounds (SBC) and microencapsulation efficiency (ME). The analysis of the TBC of the microencapsulates, referred to as aggregate content of both encapsulated and adsorbed polyphenolic compounds in the micropowders, was performed according to Ricci et al. [19], with minor modifications. Briefly, the microcapsules were disrupted by adding 25 mg of the powder into 1 mL of a methanol/acetic acid/water solution (50:8:42 *v*/*v*/*v*). Microcapsules were dissolved by vortexing (1 min) followed by ultrasonication (20 min). The vortex and ultrasonication procedures were repeated twice. The samples were then centrifuged at 14,500 rpm for 5 min, and then the supernatant was collected and filtered using a 0.22 µm cellulose acetate syringe filter. The total amount of polyphenolic compounds was quantified using the Folin–Ciocalteu method described in Section 4.4, and the results were expressed as mg GAE per gram of microencapsulated powder. For the determination of bioactive compounds adsorbed on the surface of the microcapsule (SBC), 25 mg of powder was added to 1 mL of an ethanol and methanol mixture (1:1 *v*/*v*). The samples were stirred for 1 min and then filtered using a 0.22 µm cellulose acetate syringe filter.

The total amount of polyphenolic compounds as well as the amount of surface polyphenolic compounds were quantified using the Folin–Ciocalteu method described in Section 2.4, and the results were expressed as mg GAE per gram of microencapsulated powder.

The following equations were applied to obtain the percentages of SBC and ME:

SBC (%) = (SBC/TBC) × 100(5)

ME (%) = 100 − SBC (%)(6)

### 4.7. Scanning Electron Microscopy (SEM)

The observation of microparticle surface morphology was carried out using a field emission scanning electron microscope (Field Emission SEM Inspect F50, FEI Company, Hillsboro, OR, USA). Microparticles were covered with a 10 nm gold film using a Sputter Coater (Cressington model 108, Ted Pella Inc., Redding, CA, USA) equipped with a thickness controller (Cressington MTM-20).

### 4.8. Cell Culture Conditions

Caco-2 cells, a human colon adenocarcinoma cell line, were obtained from the American Type Culture Collection (ATCC HTB-37, Rockville, MD, USA) and maintained according to ATCC protocols. The cells, between passages 15 to 25 for all experiments, were cultured in DMEM, Dulbecco’s Modified Eagle Medium (Thermo Fisher Scientific Inc., Waltham, MA, USA). Cells were maintained in culture using 10% fetal bovine serum (Biowest USA Inc., Lakewood Ranch, Bradenton, FL, USA), 1% antibiotics, and 1% MEM non-essential amino acid solution (Corning; Mediatech, Inc., Manassas, VA, USA) at 37 °C in a humidified atmosphere of 5% CO_2_/95% air. When cells reached nearly 90% confluence, they were trypsinized and subcultured onto plates for subsequent experiments.

### 4.9. Metabolic Activity and Cellular Antioxidant Capacity

First, the MTT-reducing capacity of the Caco-2 cells was evaluated as a marker of metabolic activity and therefore an estimation of cytotoxicity. When cells reached 90% confluence, they were trypsinized and seeded at a density of 50,000 cells per well in a 96-well plate (in 200 μL of culture medium per well). These experiments were carried out as described by Ospina-Posada et al. [51]. After 24 h, the culture medium was discarded, and the cells were washed twice with 1X PBS and subsequently treated for a metabolic activity assay or to assess their cellular antioxidant capacity.

The treatments selected to test the metabolic activity and cellular antioxidant capacity were the following: EET-80, EET-80 microencapsulated with maltodextrin in a 1:50 ratio, maltodextrin, 0.5% DMSO as a resuspension vehicle of the extract, and PBS as a positive control. EET-80 was dissolved in pure DMSO, with subsequent dilutions performed with a 25 mM sodium phosphate buffer at pH 7.4 until reaching a final concentration of 0.5% DMSO within the well.

To evaluate cellular metabolic activity, the reduction of MTT was used as a marker [51]. To evaluate acute local events, after 40 min of treatment, the cells were washed with PBS, and after, MTT (20 µL of 2.5 mg/mL) and PBS (80 µL) were added to each well. The Caco-2 cells were incubated again (150 min, 37 °C, 5% CO_2_/95% air). The supernatant was removed and DMSO (100 µL) was added to dissolve the formed formazan. After incubation (10 min at 37 °C), the absorbance of the adhered cells in 1X PBS was read at 540 nm in a microplate reader (Infinite pro 200, Austria GmbH, Groedig, Austria). The results were expressed as a percentage of MTT reduction and given in %, where 100% of the metabolic activity consisted of PBS-treated Caco-2 cells (positive control).

To assess the cellular antioxidant capacity, DCFH-DA was used as a fluorescent probe to ROS [52,53]. Briefly, after 24 h from seeding, cells were loaded with DCFH-DA (50 μM) for 20 min, followed by washing with PBS and subsequent exposure to the described treatments for 40 min. Cells were then washed with PBS to remove remaining samples, and fluorescence in adhered cells was measured in 1X PBS (excitation 485 nm/emission 530 nm) in a microplate reader (Infinite pro 200, Austria GmbH, Groedig, Austria). The value of 100% of intracellular oxidative stress was attributed to cells treated with 5 mM H_2_O_2_, corresponding to the difference resulting from subtracting the fluorescence emitted by cells with hydrogen peroxide and the fluorescence emitted by cells exposed to PBS. The percentage of intracellular oxidative stress (IOS) attributed to the conditions studied was calculated by subtracting the fluorescence emitted by cells treated with PBS from the fluorescence emitted by cells treated with different concentrations of EET-80, microencapsulated EET-80, maltodextrin, or 0.5% DMSO and dividing the result by the subtraction of the fluorescence emitted by cells treated with H_2_O_2_ minus the fluorescence with cells treated with PBS.

### 4.10. Statistical Analysis

All measurements, if not differently stated, were performed in triplicates. Statistical analyses were carried out using Prism Version 10.4.1 (GraphPad Software, San Diego, CA, USA). One-way ANOVA and Tukey’s multiple comparison test were used to analyze the data considering *p* ≤ 0.05.

## 5. Conclusions

This study demonstrated that grape pomace from the Carménère variety—a major by-product of the Chilean wine industry—is a valuable source of phenolic compounds with a high antioxidant capacity. The application of hydroalcoholic extractions using ethanol–water mixtures enabled selective recovery of polyphenols depending on solvent polarity. The 50% ethanol extract (EET-50) presented the most balanced performance in terms of extraction yield, total phenolic content, and chemical profile, while the 80% ethanol extract (EET-80) showed the highest anthocyanin content and the greatest antioxidant capacity, particularly in FRAP and ORAC assays.

EET-80 was selected for spray-drying microencapsulation using maltodextrin, which resulted in high encapsulation yields (>73%) and efficiencies (>80%), especially in the 1:50 extract-to-carrier formulation. This formulation exhibited favorable particle size distribution and color stability and preserved antioxidant properties. In vitro assays using undifferentiated Caco-2 cells confirmed the biocompatibility of the microencapsulated extract and its capacity to significantly reduce intracellular oxidative stress, outperforming the free extract in cellular protection.

Altogether, these findings reinforce the potential of Carménère grape pomace as a high-value functional ingredient. As a sub-product of the winemaking process, this material aligns with the principles of the circular economy, offering a sustainable pathway to reduce agro-industrial waste while generating innovative, health-oriented products. The integration of green extraction and microencapsulation technologies provides a scalable solution to transform this residue into stable, bioactive powders suitable for incorporation into functional foods or nutraceutical formulations.

Future studies should address the release kinetics, gastrointestinal stability, and bioavailability of the encapsulated phenolics and validate their health-promoting effects in differentiated intestinal models, 3D cell cultures, and in vivo systems. These evaluations will be essential to guide the development of effective, evidence-based applications for intestinal health and beyond.

## Figures and Tables

**Table 1:** Solvent extraction mixtures, pH, and yields of extracts obtained from Carménère grape pomace through dynamic maceration (50 °C; 90 min; 250 rpm agitation).

Extract	Extraction Solvent (Ethanol–Water)	pH	Yield According to Initial Dry Mass (%)
EGA	0/100	3.80 ± 0.10	13.75 ± 2.42 ^a^
EET-25	25/75	4.00 ± 0.10	22.03 ± 0.05 ^b^
EET-50	50/50	4.35 ± 0.13	15.10 ± 1.50 ^a^
EET-80	80/20	4.55 ± 0.06	13.67 ± 0.87 ^a^
EGE	100/0	5.00 ± 0.20	13.32 ± 0.81 ^a^

Different letters indicate significant differences between samples (*p* < 0.05, calculated using one-way ANOVA and Tukey’s multiple comparison test). Results are presented as mean ± standard deviation.

**Table 2:** Quantification of individual phenolic compounds (µg/g dw) in grape pomace extracts obtained with different ethanol–water ratios, determined by HPLC-DAD.

Polyphenols	EGA	EET-25	EET-50	EET-80	EGE
Phenolics acids (µg/g dw)					
Gallic Acid	365.55 ± 3.01 ^a^	334.14 ± 7.14 ^a^	578.89 ± 7.02 ^b^	478.99 ± 4.01 ^c^	587.22 ± 14.74 ^b^
Vanillic Acid	ND	ND	216.33 ± 1.25	ND	ND
Flavonoids and derivatives (µg/g dw)					
Catechin	34.15 ± 1.16 ^a^	474.47 ± 41.28 ^b^	581.12 ± 30.09 ^c^	437.11 ± 16.31 ^b^	69.90 ± 6.66 ^d^
Epicatechin	71.43 ± 5.89 ^a^	1361.11 ± 80.43 ^b^	1788.33 ± 218.66 ^c^	1365.41 ± 107.15 ^b^	2670.36 ± 114.54 ^d^
Epigallocatechin Gallate	n.d	n.d	15.13 ± 0.41 ^a^	n.d	9.12 ± 0.50 ^b^
Epicatechin Gallate	n.d	0.084 ± 0.01 ^a^	0.777 ± 0.01 ^b^	0.327 ± 0.01 ^c^	1.009 ± 0.03 ^d^
Kaempferol	n.d	0.013 ± 0.01 ^a^	0.097 ± 0.01 ^b^	0.061 ± 0.01 ^c^	0.097 ± 0.01 ^b^
Kaempferol 3-rutinoside	n.d	0.278 ± 0.02 ^a^	0.568 ± 0.16 ^b^	0.489 ± 0.05 ^c^	n.d
Quercetin	0.10 ± 0.01 ^a^	0.17 ± 0.01 ^b^	0.60 ± 0.01 ^c^	0.66 ± 0.02 ^d^	0.50 ± 0.02 ^e^
Taxifolin	n.d	n.d	n.d	0.013 ± 0.01	n.d
Procyanidins (µg/g dw)					
Procyanidin B1	n.d	0.017 ± 0.01 ^a^	n.d	n.d	0.014 ± 0.01 ^a^
Anthocyanins (µg/g dw)					
Malvidin-3-*O*-glucoside	27.19 ± 1.59 ^a^	66.33 ± 2.22 ^b^	106.70 ± 5.64 ^c^	118.04 ± 4.68 ^d^	15.16 ± 0.68 ^e^

Results are expressed in µg per gram of dry extract weight (µg/g dw) and presented as mean ± SD (*n* = 3). n.d: not detected. Different superscript letters (^a,b,c,d,e^) within a row indicate statistically significant differences in compound quantification among the five extracts analyzed, as determined by one-way ANOVA followed by Tukey’s post hoc test (*p* < 0.05).

**Table 3:** Antioxidant capacity of grape pomace extracts obtained with different ethanol–water ratios, evaluated by DPPH, FRAP, and ORAC-FL assays, as well as IC_50_ values.

Antioxidant Capacity	EGA	EET-25	EET-50	EET-80	EGE	Trolox
DPPH (µmol TE/g dw)	76.99 ± 2.92 ^a^	347.22 ± 19.01 ^b^	985.03 ± 47.04 ^c^	884.43 ± 41.31 ^d^	348.45 ± 7.37 ^e^	-
IC_50_ (µg/mL)	229.60 ± 8.75 ^a^	50.96 ± 2.86 ^b^	17.96 ± 0.87 ^c^	20.00 ± 0.92 ^c^	50.70 ± 1.08 ^b^	4.42 ± 0.04 ^d^
FRAP (µmol TE/g dw)	330.10 ± 11.12 ^a^	843.21 ± 22.89 ^b^	1587.12 ± 44.52 ^c^	2909.33 ± 37.55 ^d^	950.93 ± 125.21 ^e^	-
ORAC-FL (µmol TE/g dw)	330.16 ± 5.82 ^a^	841.34 ± 112.23 ^b^	1571.26 ± 138.71 ^c^	1864.32 ± 157.8 ^d^	792.41 ± 29.5 ^e^	-

Results are expressed as µmol Trolox equivalents (TE) per gram of dry weight (dw) for DPPH, FRAP, and ORAC-FL, and as µg/mL for IC_50_. Values are presented as mean ± SD (*n* = 3). Different superscript letters (^a,b,c,d,e^) within a row indicate statistically significant differences between samples, as determined by one-way ANOVA followed by Tukey’s post hoc test (*p* < 0.05).

**Table 4:** Characterization of the spray-dried powder obtained from the microencapsulation of EET-80 with maltodextrin in different proportions.

Sample	Extract/ MD Ratio	Powder Color (CIE L*, a*, b*)				Encapsulation Yield (%)	TBC (mg EAG/g)		ME (%)	Average Size (µm) (Dx90)
		L*	a*	b*	Color					
EET-80	1:10	71.55 ± 0.22	5.16 ± 0.01	−3.73 ± 0.01	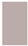	73.16	117.46 ± 2.23 ^a^	14.71 ± 0.32 ^a^	87.47 ± 0.03 ^a^	3.31 ± 1.32 ^a^
	1:50	78.17 ± 0.15	6.95 ± 0.03	−2.63 ± 0.01	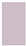	81.54	168.96 ± 1.88 ^b^	24.05 ± 1.48 ^b^	85.72 ± 0.72 ^a^	4.03 ± 1.68 ^a^
	1:100	83.13 ± 0.12	3.96 ± 0.01	−1.81 ± 0.02	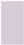	82.34	171.83 ± 4.88 ^b^	34.74 ± 1.24 ^c^	80.88 ± 1.20 ^b^	5.14 ± 1.69 ^a^

Different superscript letters within a column indicate statistically significant differences between samples, as determined by one-way ANOVA followed by Tukey’s post hoc test (*p* < 0.05). The measured coordinates (L*, a*, b*) were converted to RGB using the online tool Color Designer (https://colordesigner.io/convert/labtorgb, accessed on 30 July 2025) and the corresponding color representations were included as visual references. Legend: TBC: total bioactive compounds; SBC: surface bioactive compounds; ME: microencapsulation efficiency. Different superscript letters (^a,b,c^) within a column indicate statistically significant differences between samples.

## Data Availability

Data are available on request from the authors.
